# Human Capital Mediates Natural Selection in Contemporary Humans

**DOI:** 10.1007/s10519-022-10107-w

**Published:** 2022-07-06

**Authors:** David Hugh-Jones, Abdel Abdellaoui

**Affiliations:** 1 School of Economics, University of East Anglia, Norwich, UK; 2 Department of Psychiatry, Amsterdam UMC, University of Amsterdam, Amsterdam, The Netherlands

**Keywords:** Natural selection, Polygenic scores, Economic theory of fertility

## Abstract

Natural selection has been documented in contemporary humans, but little is known about the mechanisms behind it. We test for natural selection through the association between 33 polygenic scores and fertility, across two generations, using data from UK Biobank (N = 409,629 British subjects with European ancestry). Consistently over time, polygenic scores that predict higher earnings, education and health also predict lower fertility. Selection effects are concentrated among lower SES groups, younger parents, people with more lifetime sexual partners, and people not living with a partner. The direction of natural selection is reversed among older parents, or after controlling for age at first live birth. These patterns are in line with the economic theory of fertility, in which earnings-increasing human capital may either increase or decrease fertility via income and substitution effects in the labour market. Studying natural selection can help us understand the genetic architecture of health outcomes: we find evidence in modern day Great Britain for multiple natural selection pressures that vary between subgroups in the direction and strength of their effects, that are strongly related to the socio-economic system, and that may contribute to health inequalities across income groups.

## Introduction

Living organisms evolve through natural selection, in which allele frequencies change in the population through differential reproduction rates. Studying the mechanisms behind natural selection can help us better understand how individual differences in complex traits and disease risk arise (Benton et al. 2021). Recent work confirms that natural selection is taking place in modern human populations, using genome-wide analysis (Barban et al. 2016; Beauchamp 2016; Conley et al. 2016; Kong et al. 2017; Sanjak et al. 2018; Fieder and Huber 2022). In particular, genetic variants associated with higher educational attainment are being selected against, although effect sizes appear small.

As yet we know little about the social mechanisms behind natural selection. The economic theory of fertility (Becker 1960) offers a potential explanation. Higher potential earnings have two opposite effects on fertility: a fertility-increasing *income effect* (higher income makes children more affordable), and a fertility-lowering *substitution effect* (time spent on childrearing has a higher cost in foregone earnings). Thus, an individual’s *human capital* – skills and personality traits which are valuable in labour markets – can increase or decrease their fertility. Genetic variants which are linked to human capital will then be selected for or against. Also, the economic theory predicts that the relative strength of income and substitution effects will vary systematically across different social groups.

This study uses data from UK Biobank (Bycroft et al. 2018) to learn more about contemporary natural selection. We test for natural selection on 33 different polygenic scores by estimating their correlation with fertility. We extend the analysis over two generations, using data on respondents’ number of siblings as well as their number of children. This is interesting because consistent natural selection over multiple generations could lead to substantive effects in the long run. Next, we examine correlations with fertility in different subgroups. Across the board, selection effects are stronger in groups with lower income and less education, among younger parents, people not living with a partner, and people with more lifetime sexual partners. Outside these groups, effects are weaker and often statistically insignificant. In some subgroups, the direction of selection is even reversed.

We then show that a simple model of human capital, education and fertility choices can give rise to these empirical results. At higher incomes, the income and substitution effects are balanced, while among lower-income people, or single parents who face a bigger time burden from childcare, the substitution effect dominates. The theory predicts that polygenic scores’ correlation with fertility is associated with their correlation with education and earnings, and we confirm this. We then run a mediation analysis, which shows that part of the correlation with fertility is indeed mediated by educational attainment. Thus, contemporary natural selection on polygenic scores can be explained by scores’ correlation with earnings-increasing human capital.

Lastly, we discuss the effects of natural selection. While our estimated effects on measured polygenic scores are small, natural selection substantially increases the correlation between polygenic scores and income, increasing genetic differences between different social groups, and thus making the “genetic lottery” (Harden 2021) more unfair.

## Results

**Fig. 1:**
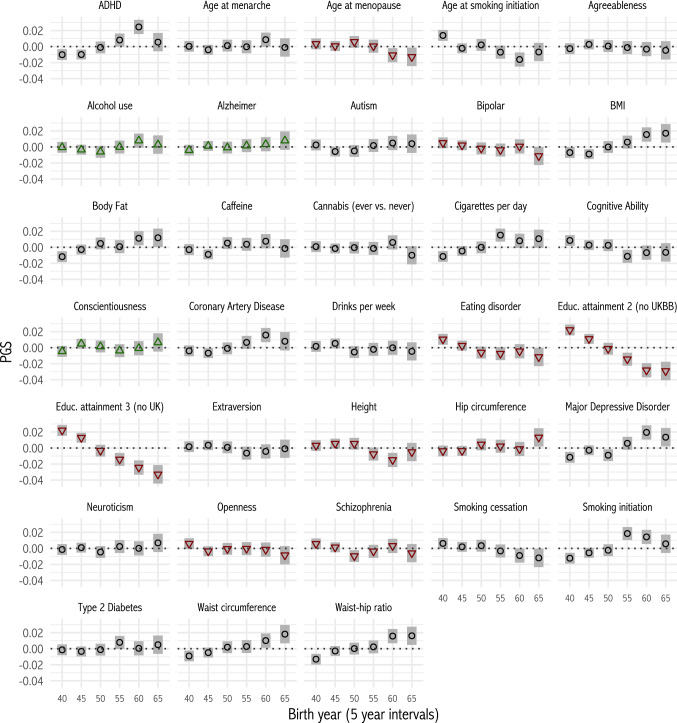
Mean polygenic scores (PGS) by birth year in UK Biobank. Symbols show means for 5-year intervals. Bars are 95% confidence intervals. Triangles denote a significant linear increase or decrease over time (p < 0.05/33)

We created polygenic scores for 33 traits in 409,629 individuals of European descent, corrected for ancestry using 100 genetic principal components (see Materials and Methods). Figure 1 plots mean polygenic scores in the sample by 5-year birth intervals. Several scores show consistent increases or declines over this 30-year period, of the order of 5% of a standard deviation. These changes could reflect natural selection within the UK population, but also emigration, or ascertainment bias in the sample (Fry et al. 2017).

To test for natural selection more directly, we regress respondents’ relative lifetime reproductive success (RLRS) on each polygenic score (PGS):

1 $$\begin{aligned} \mathrm {RLRS}_i = \alpha + \beta \mathrm {PGS}_i + \varepsilon _i \end{aligned}$$

RLRS is defined as respondent *i*’s number of children, divided by the mean number of children of people born in the same year. The “selection effect”, $\beta$, reflects the strength of natural selection within the sample. In fact, since polygenic scores are normalized, $\beta$ is the expected polygenic score among children of the sample (Beauchamp 2016).^1^ Note that equation (1) does not control for many environmental and genetic factors that could affect fertility, and as a result, $\beta$ is not an estimate of the causal effect of a polygenic score on fertility. However, natural selection is a matter of correlation not causation: polygenic scores which correlate with high fertility are being selected for, whatever the underlying causal mechanism.

**Fig. 2:**
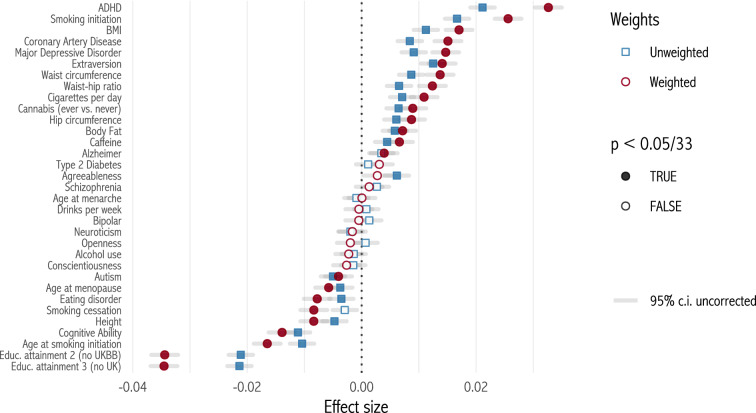
Selection effects: weighted and unweighted regressions. Each point represents a single bivariate regression of RLRS on a polygenic score. P value threshold is 0.05, Bonferroni-corrected for multiple comparisons. Confidence intervals are uncorrected

Figure 2 plots selection effects in the whole sample.^2^ To correct for ascertainment bias, we use participant weights from Alten et al. (2022), which match the UK Biobank eligible population on sex, birth year, location, education, employment, health, household size and tenure, number of cars and age at death. Weighting makes a large difference: effect sizes go up by a mean of 48%.^3^ 23 out of 33 weighted selection effects are significant at *p* < 0.05/33.

We now show the empirical puzzles which motivate our economic model. Each concerns differences in the strength of natural selection across different subgroups in the sample. We re-estimate (1) splitting the sample by demographic and social variables, including income and education, and family structure variables including age at first live birth, presence of a partner, and lifetime number of sexual partners.

**Fig. 3:**
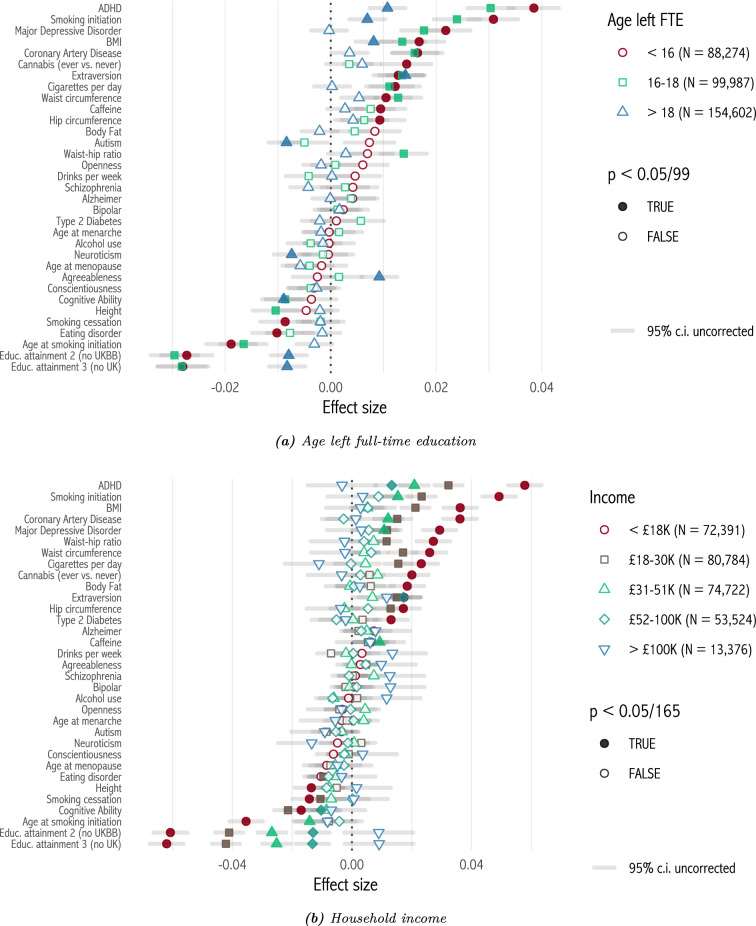
Selection effects by education and income

**Fig. 4:**
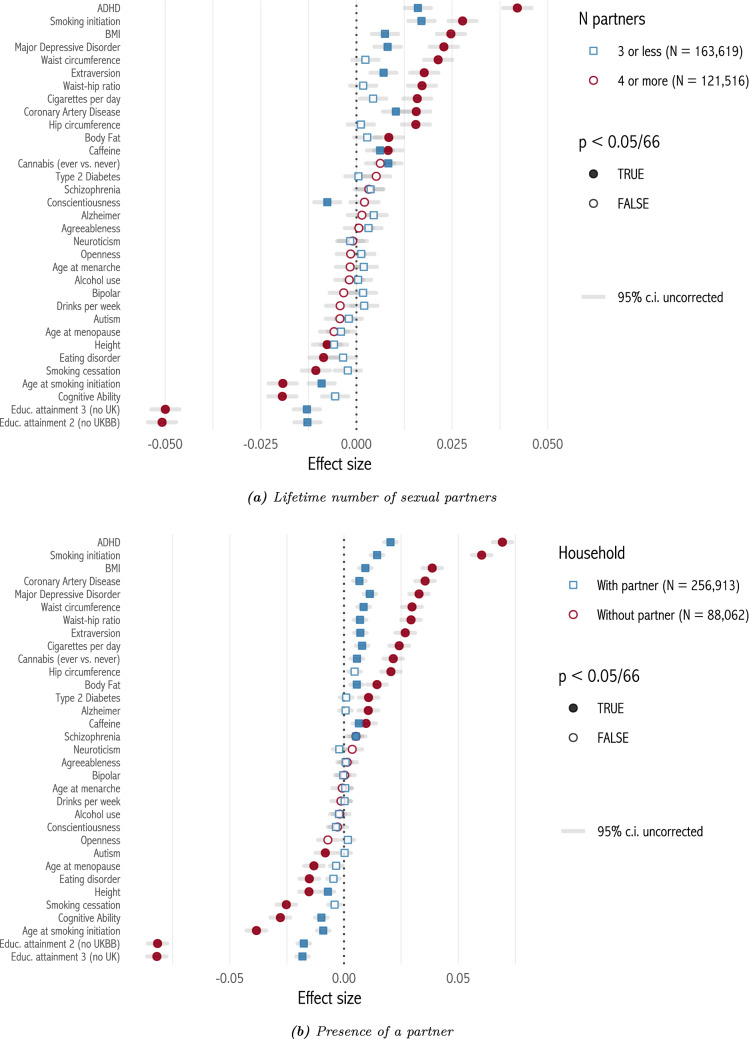
Selection effects by number of sexual partners and presence of a partner

Figure 3 plots selection effects for each polygenic score, grouping respondents by age of completing full-time education, and by household income. Effects are larger and more significant for the lowest education category, and for the lowest income category. The median percentage difference between the lowest and highest education categories, among scores which are significant for the lowest category and have the same sign across categories, is 249%. Between the lowest and highest income categories, it is 595%. These results are robust to controlling for respondents’ age (Appendix sect. 8.4). Turning to family structure, we split respondents by lifetime number of sexual partners, at the median value of 3 (Fig. 4a). Now, selection effects are larger and more significant among those with more than 3 lifetime partners, with a median percentage difference of 191%. Next we split respondents by whether they were living with a spouse or partner at the time of interview (Figure 4b). Effects are larger among those not living with a spouse or partner. The median percentage difference is 281%.^4^

**Fig. 5:**
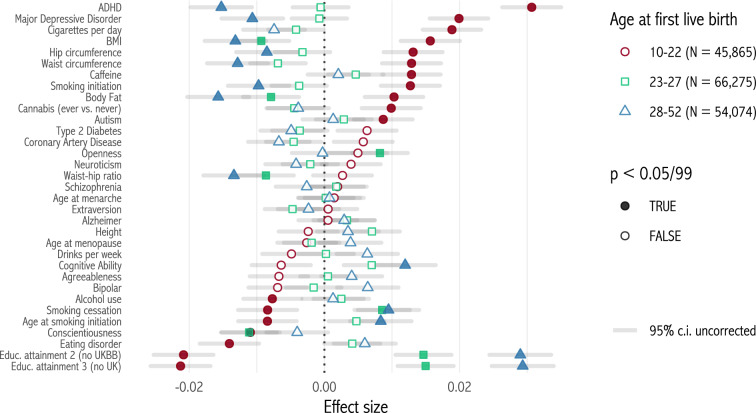
Selection effects by age at first live birth terciles (women only)

Lastly, we split female respondents by age at first live birth (AFLB).^5^ There is evidence for genetic effects on AFLB (Barban et al. 2016), and there is a close link between this variable and number of children born. Figure 5 shows effect sizes estimated separately for each tercile of AFLB. Effects are strikingly different across terciles. Educational attainment, ADHD and MDD are selected for amongst the youngest third of mothers, but selected against among the oldest two-thirds. Similarly, several polygenic scores for body measurements are selected against only among older mothers. The correlation between effect sizes for the youngest and oldest terciles is –0.83. To investigate this further, we estimate equation (1) among females, *controlling* for AFLB. In 18 out of 33 cases, effects change sign when controls are added. The correlation between effect sizes controlling for AFLB, and raw effect sizes, is –0.58. Thus, selection effects seem to come through two opposing channels: a correlation with AFLB, and an opposite-signed correlation with number of children after AFLB is controlled for.

We emphasize that these categories are not exogenous to polygenic scores. For example – both in the data (Appendix Fig. 17) and in our theoretical model – education and age at first live birth are choice variables, which are endogenous to a person’s human capital and to relevant polygenic scores. Nevertheless, differences in selection effects across subgroups constrain the set of possible explanations. A good theory of contemporary natural selection needs to show how these differences come about. As we describe below, a model based on the economic theory of fertility can do just that.

We also examine selection effects among respondents’ parents, using information on respondents’ number of siblings to calculate parents’ RLRS. Effect sizes of polygenic scores are highly correlated across the two generations (Appendix Fig. 12). Median-splitting respondents by year of birth, we find little evidence of change in effect sizes among the parents’ generation. There is some evidence that selection effect sizes are increasing in the respondents’ generation, with 8 polygenic scores showing a significant increase. We also check whether selection effects vary by AFLB and socio-economic status in the parents’ generation, using the 1971 Townsend deprivation score of respondents’ birthplace as a proxy for income (Townsend 1987). Results show the same pattern as for the respondents’ generation. Effect sizes are larger and more often significant in the most deprived areas (Appendix Fig. 13). Effects are larger among younger fathers and mothers, and change sign when controlling for AFLB (Appendix Figs 15, 16). Lastly, we check for a “quantity-quality tradeoff” between parents’ number of children and number of grandchildren. We don’t find any: in fact, the correlation between respondents’ and parents’ RLRS is positive ($\rho$ = 0.1, $p < 2 \times 10^{-16}$).

## Human Capital and Natural Selection

These results show that selection effects are weaker, absent, or even reversed among some subgroups of the population. A possible explanation for this comes from the economic theory of fertility (Becker 1960; Willis 1973; Becker and Tomes 1976). According to this theory, increases in a person’s wage affect their fertility via two opposing channels. There is an *income effect* by which children become more affordable, like any other good. There is also a *substitution effect*: since childrearing has a cost in time, the opportunity cost of childrearing increases if one’s market wage is higher. The income effect leads higher earners to have more children. The substitution effect leads them to have fewer.

Suppose that certain genetic variants correlate with *human capital*: skills or other characteristics that affect an individual’s earnings in the labour market (Mincer 1958; Becker 1964). These variants may then be associated with opposing effects on fertility. The income effect will lead to natural selection in favour of earnings-increasing variants (or variants that are merely associated with higher earnings). The substitution effect will do the reverse.

To show this, consider a simple model of fertility choices. *h* is an individual’s level of human capital. For now, we simply identify this with his or her wage *W*. Raising a child takes time *b*. People maximize utility *U* from the number of children *N* and from income $Y\equiv (1-bN)W$:

$$\begin{aligned} U = u(Y)+aN. \end{aligned}$$

Here *a* captures the strength of preference for children. $u(\cdot )$ captures the taste for income, and is increasing and concave. We treat *N* as continuous, in line with the literature: this can be thought of as the expected number of children among people with a given *a*, *b* and *W*. The marginal benefit of an extra child is $\frac{dU}{dN} = -bWu'(Y)+a$. The effect of an increase in human capital on this marginal benefit is

$$\begin{aligned} \frac{d^{2}U}{dNdW}=\underbrace{-bu'(Y)}_{\text {Substitution effect}}\underbrace{-bYu''(Y)}_{\text {Income effect}}. \end{aligned}$$

The *substitution effect* is negative and reflects that when wages increase, time devoted to childcare costs more in foregone income. The positive *income effect* depends on the curvature of the utility function, and reflects that when income is higher, the marginal loss of income from children is less painful.

To examine education and fertility timing, we extend the model to two periods. For convenience we ignore time discounting, and assume that credit markets are imperfect so that agents cannot borrow. Write

2 $$\begin{aligned} U(N_{1},N_{2}) = u(Y_{1}) + u(Y_{2}) + aN_{1} + aN_{2} \end{aligned}$$

Instead of identifying human capital with wages, we now allow individuals to spend time $s \in [0,1]$ on education in period 1. Education is complementary to human capital $h > 0$, and increases period 2 wages, which take the simple functional form $w(s,h) = sh$. We normalize period 1 wages to 1, and let $u(\cdot )$ take the constant relative risk aversion form $u(y)=\frac{y^{1 - \sigma } - 1}{1 - \sigma }$. $\sigma > 0$ measures the curvature of the utility function, i.e. the decline in marginal utility of income as income increases. We examine total fertility $N^{*} = N_{1}^{*} + N_{2}^{*}$ and the *fertility-human capital relationship*, $\frac{dN^{*}}{dh}$. For $\sigma < 1$ and close enough to 1, Table 1 shows five theoretical predictions, along with our corresponding empirical results for the correlation between polygenic scores and RLRS.^6^ The key insight of the model is that for middling levels of $\sigma$, the substitution effect dominates at low income levels, but as income increases, the income and substitution effect balance out.

**Table 1:** Predictions from the theoretical model and corresponding empirical results

	Theory: the fertility-human capital relationship is.	Empirical results
1.	Negative: $\frac{dN^{*}}{dh} < 0$.	Figures 1 and 2.
2.	Weaker (closer to zero) at higher wages and/or levels of human capital.	Figure 3a. Selection effects are also weaker at higher polygenic scores for educational attainment (Appendix Fig. 10).
3.	More negative when the time burden of children *b* is larger.	Stronger effects for single parents (Fig. 4).
4.	Weaker at higher levels of education *s*.	Figure 3b.
5.	Weaker among those who start fertility in period 2 ($N_{1}^{*} = 0$) than among those who start fertility in period 1 ($N_{1}^{*} > 0$).	Effects weaker among those starting fertility later (Fig. 5).

Thus, a simple economic model can explain many of our results. Other empirical work in economics also supports the link from human capital to fertility. Caucutt et al. (2002) and Monstad et al. (2008) show that education and skills affect age at first birth and fertility. Income decreases fertility at low income levels, but increases it at higher income levels (Cohen et al. 2013). US fertility decreases faster with education among single mothers than married mothers (Baudin et al. 2015), in line with our prediction 3 and as predicted by Becker (1981). A related literature shows negative correlations between IQ and fertility (e.g. Lynn and Van Court 2004; Reeve et al. 2018).

**Fig. 6:**
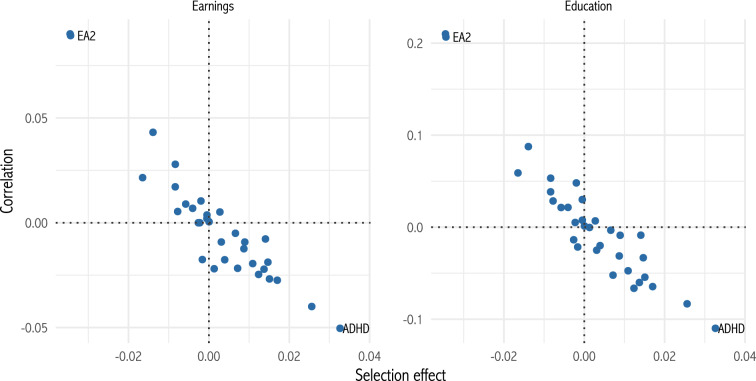
Selection effects by correlations with earnings and educational attainment. Each point represents one polygenic score. Selected scores are annotated

## Testing the Theory

We test the economic theory in two ways. First, it predicts that genetic variants will be selected for (or against) in proportion to their correlation with human capital. Figure 6 plots selection effects on each polygenic score against that score’s correlation with two measures of human capital: earnings in a respondent’s first job, and educational attainment. The relationships are strongly negative. Thus, human capital appears to be relevant to natural selection. The negative relationship suggests that substitution effects dominate income effects, which fits the known negative association between income and fertility (Becker 1960; Jones and Tertilt 2006). The correlations reverse when we control for age at first live birth, suggesting that within AFLB categories, the income effect dominates.

**Fig. 7:**
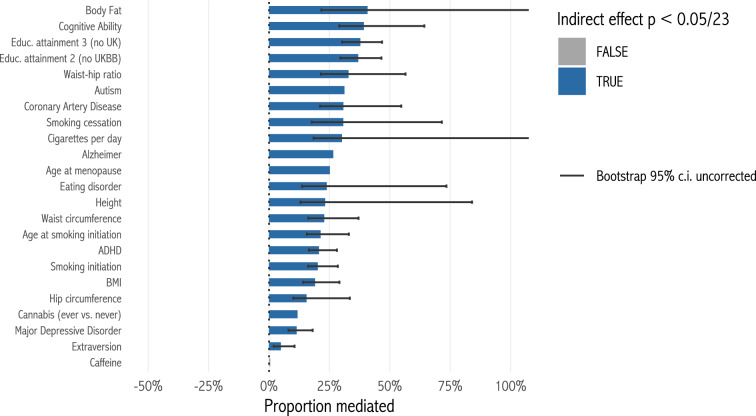
Proportion of selection effect mediated by educational attainment, among polygenic scores with significant selection effects. Bootstrap confidence intervals for the proportion are shown only where the interval is bounded (Franz 2007)

Second, we run a mediation analysis to directly test whether the correlation between each polygenic score and fertility is mediated by educational attainment (Appendix Table 4). We use the 23 scores where the selection effect is significant at *p* < 0.05/33. Figure 7 shows estimated proportions explained by educational attainment, along with bootstrap 95% confidence intervals (uncorrected; 100 bootstraps). For 22 scores, the indirect effect of the score on fertility *via* educational attainment takes the same sign as the overall effect, and is significantly different from zero (*p* < 0.05/23). Among these scores, the median proportion of the total effect explained by the indirect effect is 25%. The educational attainment variable is a relatively crude measure of human capital: more accurate measures would likely explain more of the total effect.

We consider three alternative theories that might explain our results. First, welfare benefits which incentivize child-bearing might be taken up more among low-income people. However, the majority of effect sizes appear unchanged over a large span of twentieth-century history (Appendix Table 3), during which government spending on child-related benefits varied considerably (Social Security Committee 1999). In general, there is only weak evidence that welfare benefits affect fertility (Gauthier 2007; see also Bergsvik et al. 2021). Future work could test this theory more explicitly. A second alternative theory is that polygenic scores correlate with the motivation to have children, i.e. parameter *a* in the model (cf. Jones et al. 2008). This theory would not explain why selection effects are smaller at higher incomes and education levels. In fact, in the model, *a*’s effect on fertility gets stronger at higher levels of human capital. A third alternative is that traits under selection are linked to externalizing behaviour and risk-seeking. This might be partially captured by our parameter $\sigma$, which can be interpreted as a measure of risk aversion over income; a more direct channel is risky sexual behaviour (Mills et al. 2021). The data here provide some support for this story: scores which might plausibly be linked to externalizing behaviour, like ADHD and younger age at smoking initiation, are selected for. However, risk-seeking seems unlikely to explain variation in fertility across the full range of scores under selection, including physical measures like waist-hip ratio and BMI. We test this theory directly by re-estimating equation (1) controlling for a measure of risk attitude (UK Biobank field 2040). The median ratio of effect sizes between regressions with and without controls is 0.98; all scores which are significant at $p < 0.05/33$ in uncontrolled regressions remain so when controlling for risk attitude. This non-result could simply reflect the imprecision of the risk attitude measure, which is a single yes/no question. But this measure *does* predict the overall number of children, highly significantly ($p < 2 \times 10^{-16}$ in 33 out of 33 regressions). Given that, and the statistical power we get from our sample size, we believe that the non-result is real: while risk attitude does predict fertility in the sample, it is not an important channel for natural selection.

## Discussion

Previous work has documented natural selection in modern populations on variants underlying polygenic traits (Beauchamp 2016; Kong et al. 2017; Sanjak et al. 2018). We show that correlations between polygenic scores and fertility are highly concentrated among specific subgroups of the population, including people with lower income, lower education, younger first parenthood, and more lifetime sexual partners. Among mothers aged 22+, selection effects are reversed. Furthermore, the size of selection effects on a polygenic score correlates with that score’s association with labour market earnings. Strikingly, some of these results were predicted by Fisher (1930), pp. 253-254. The economic theory of fertility gives a parsimonious explanation for these findings. Because of the substitution effect of earnings on fertility, scores are selected for when they correlate with low human capital, and this effect is stronger at lower levels of income and education.

Polygenic scores which correlate with lower earnings and less education are being selected for. In addition, many of the phenotypes under positive selection are linked to disease risk. Many people would probably prefer to have high educational attainment, a low risk of ADHD and major depressive disorder, and a low risk of coronary artery disease, but natural selection is pushing against genes associated with these traits. Potentially, this could increase the health burden on modern populations, but that depends on effect sizes. Our results show that naïve estimates can be affected by sample ascertainment bias. There may be remaining sources of ascertainment bias after our weighting; if so, we expect that, like the sources of ascertainment we have controlled for, they probably bias our results towards zero. Researchers should be aware of the risks of ascertainment when studying modern natural selection.

We also do not know how estimated effect sizes of natural selection will change as more accurate polygenic scores are produced, or whether genetic variants underlying other phenotypes will show a similar pattern to those studied here. Also, effects of polygenic scores may be inflated in population-based samples, because of indirect genetic effects, gene-environment correlations, and/or assortative mating (Lee et al. 2018; Selzam et al. 2019; Kong et al. 2018; Howe et al. 2021), although we do not expect that this should change their association with number of offspring, or the resulting changes in allele frequencies. Although effects on our measured polygenic scores are small even after weighting, individually small disadvantages can cumulate to create larger effects. Lastly, note that our data comes from people born before 1970. Recent evidence suggests that fertility patterns may be changing (Doepke et al. 2022). Overall, it is probably too early to tell whether modern natural selection has a substantively important effect on population averages of phenotypes under selection.

**Fig. 8:**
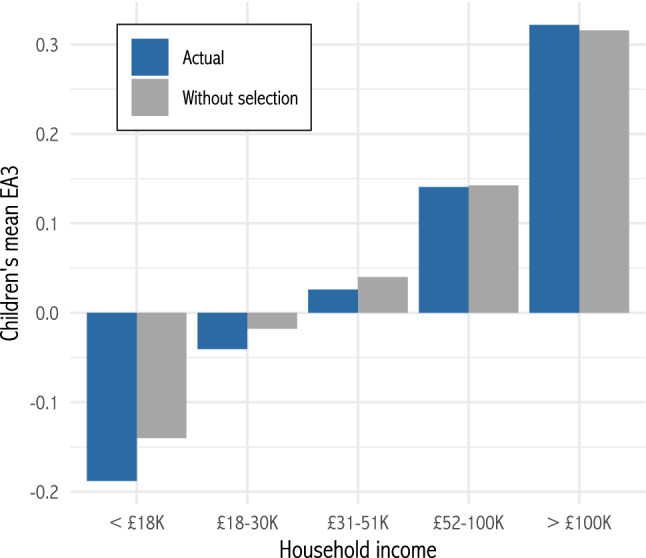
Mean polygenic score for educational attainment (EA3) of children by household income group. Blue is actual. Grey is hypothetical in the absence of selection effects (Color figure online)

Because selection effects are concentrated in lower-income groups, they may also increase inequality with respect to polygenic scores. For example, Figure 8 plots mean polygenic scores for educational attainment (EA3) among children from households of different income groups. The blue bars show the actual means, i.e. parents’ mean polygenic score weighted by number of children. The grey bars show the hypothetical means if all households had equal numbers of children. Natural selection against genes associated with educational attainment is stronger at the bottom of the income distribution, and this increases the differences between groups. Overall, natural selection increases the correlation of polygenic scores with income for 28 out of 33 polygenic scores, with a median percentage increase of 16.43% in the respondents’ generation (Appendix Table 5). If inequalities in polygenic scores are important for understanding social structure and mobility (Belsky et al. 2018; Rimfeld et al. 2018; Harden 2021), then these increases are substantive. Similarly, since many polygenic scores are predictive of disease risk, they could potentially increase health inequalities. In general, the evolutionary history of anatomically modern humans is related to disease risk (Benton et al. 2021); understanding the role of contemporary natural selection may help researchers to map the genetic architecture of health disparities.

Existing evidence on human natural selection has led some to “biocosmic pessimism” (Sarraf and Feltham 2019). Others are more sanguine, and argue that natural selection’s effects are outweighed by environmental improvements, like those underlying the Flynn effect (Flynn 1987). The evidence here may add some nuance to this debate. Patterns of natural selection have been relatively consistent across the past two generations, but they are not the outcome of a single, society-wide phenomenon. Instead they result from opposing forces, operating in different parts of society and pulling in different directions.

Any model of fertility is implicitly a model of natural selection, but so far, the economic and human genetics literatures have developed in parallel. Integrating the two could deepen our understanding of natural selection in modern societies. Economics possesses a range of theoretical models on the effects of skills, education and income (see Hotz et al. 1997; Lundberg and Pollak 2007). One perennial problem is how to test these theories in a world where education, labour and marriage markets all interact. Genetic data, such as polygenic scores, could help to pin down the direction of causality, for example via Mendelian randomization (Smith and Shah 2003). Conversely, economic theories and empirical results can shine a light on the mechanisms behind natural selection, and thereby on the nature of individual differences in complex traits and disease risk.

## Materials and Methods

We use participant data from UK Biobank (Bycroft et al. 2018), which has received ethical approval from the National Health Service North West Centre for Research Ethics Committee (reference: 11/NW/0382). We limit the sample to white British participants of European descent, as defined by genetic estimated ancestry and self-identified ethnic group, giving a sample size of 409,629. For regressions on number of children we use participants over 50 (males)/45 (females), since most fertility is completed by this age. This gives a sample size of 348,595.

Polygenic scores were chosen so as to cover a reasonably broad range of traits, and based on the availability of a large and powerful GWAS which did not include UK Biobank. Scores were computed by summing the alleles across ~1.3 million genetic variants weighted by their effect sizes as estimated in 33 genome-wide association studies (GWASs) that excluded UK Biobank. To control for population stratification, we corrected the polygenic scores for 100 principal components (PCs). To compute polygenic scores and PCs, the same procedures were followed as described in Abdellaoui et al. (2019).

Earnings in first job are estimated from mean earnings in the 2007 Annual Survey of Hours and Earnings, using the SOC 2000 job code (Biobank field 22617).

Weighting data was kindly provided by Alten et al. (2022).

## Figures and Tables

**Fig. 9:**
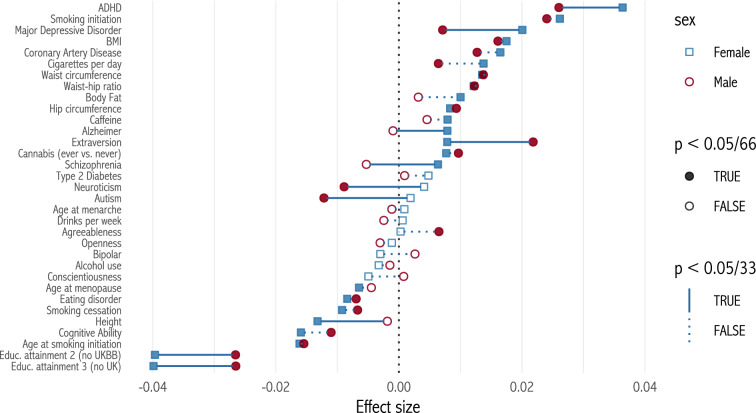
Selection effects by sex. Solid lines are significant differences at p < 0.05/33. Solid points are significantly different from 0 at p < 0.05/66

**Fig. 10:**
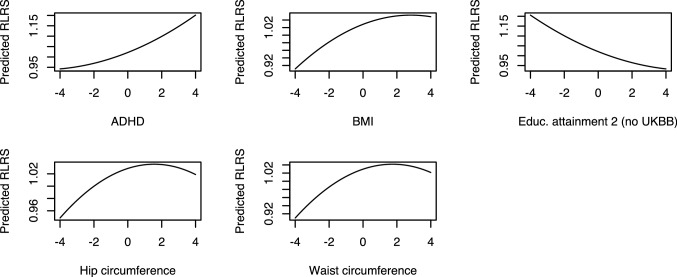
Stabilizing/disruptive selection: predicted number of children by polygenic score

**Fig. 11:**
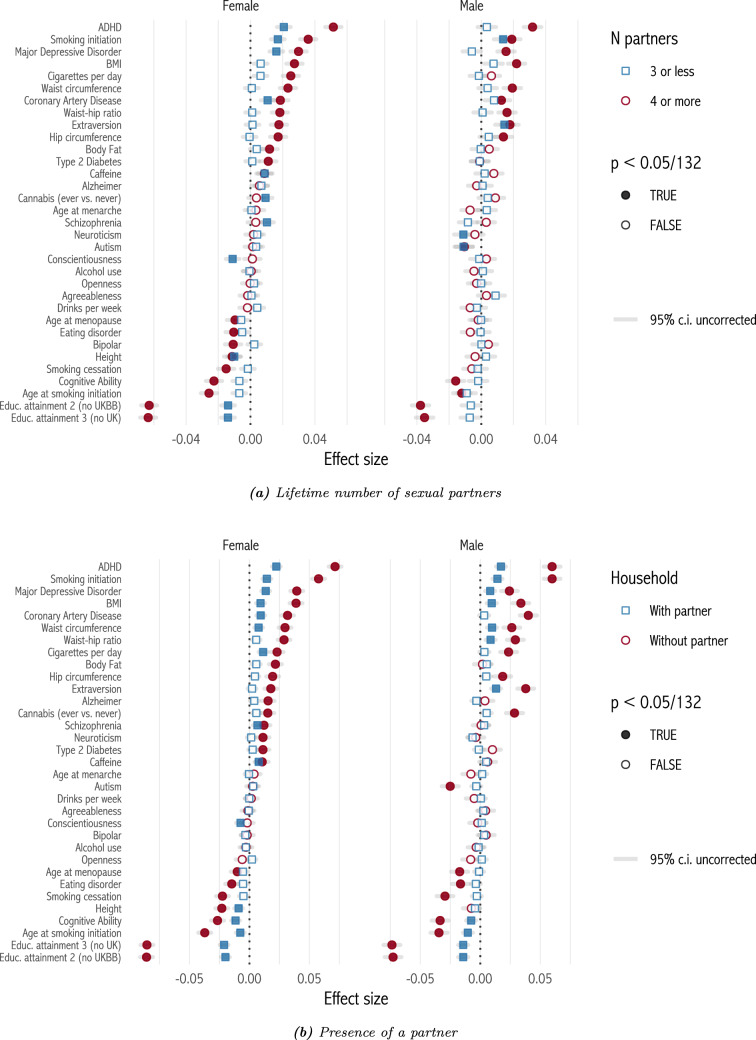
Selection effects by number of sexual partners and presence of a partner, for men and women separately

**Fig. 12:**
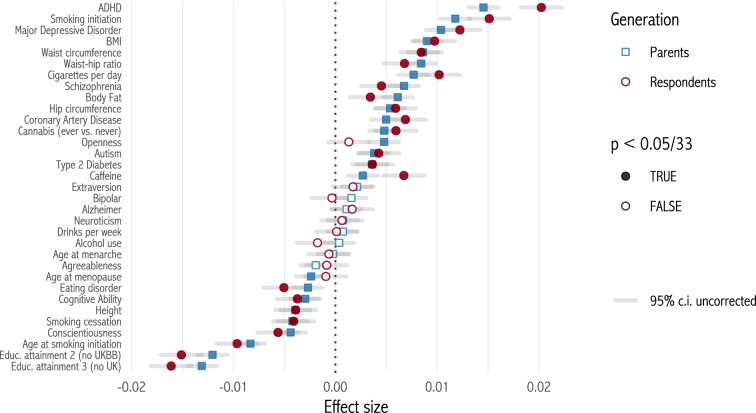
Selection effects, respondents’ parents vs. respondents. Parental generation weights multiplied by 1/number of siblings. Respondents’ regression excludes childless respondents

**Fig. 13:**
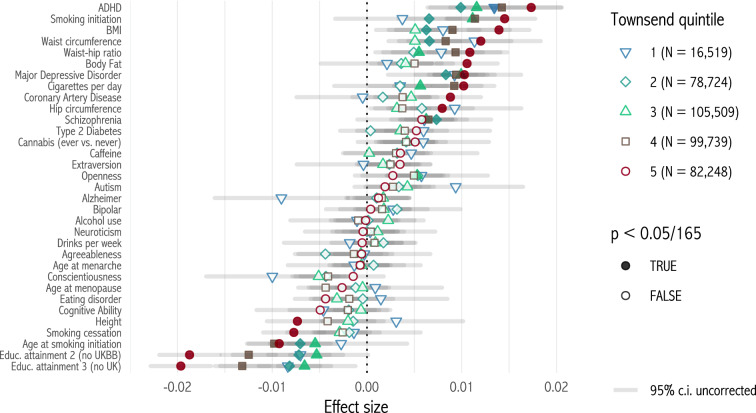
Selection effects (parents’ RLRS) by Townsend deprivation quintile of birth area. Higher = more deprived. Weights multiplied by 1/number of siblings

**Fig. 14:**
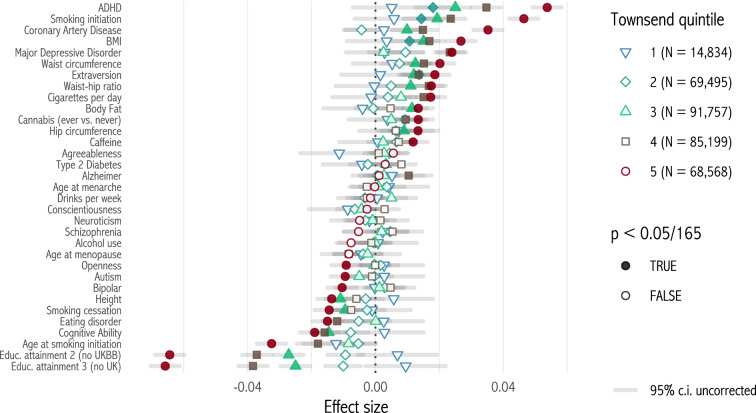
Selection effects in the respondents’ generation by Townsend deprivation quintile of birth area. Higher = more deprived

**Fig. 15:**
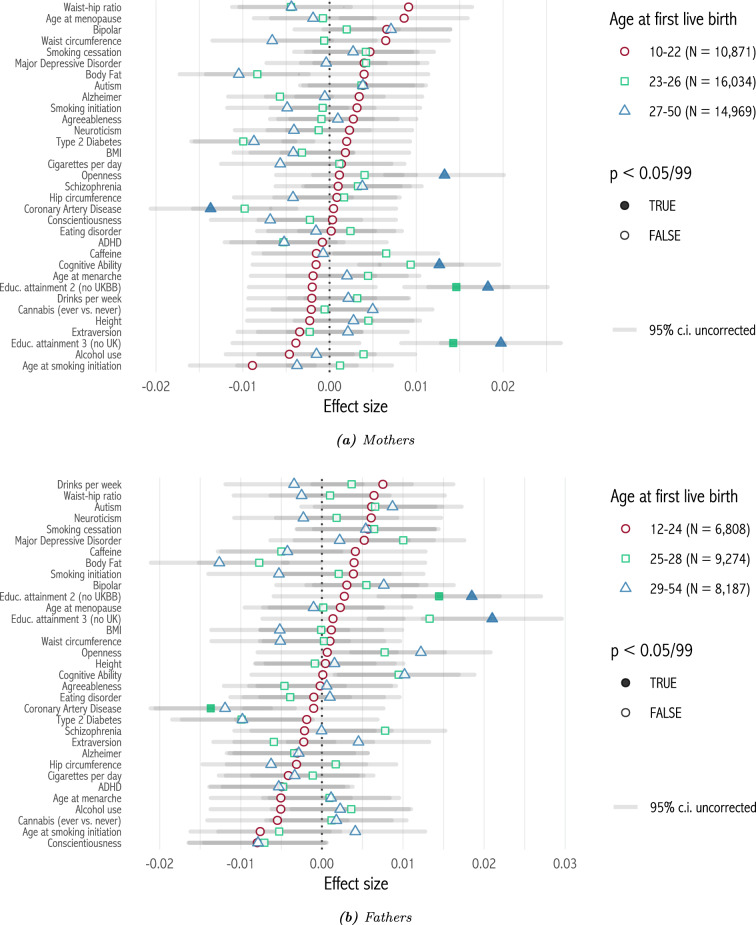
Selection effects (parents’ RLRS) among eldest siblings, by parents’ age at first live birth terciles. Weights multiplied by 1/number of siblings

**Fig. 16:**
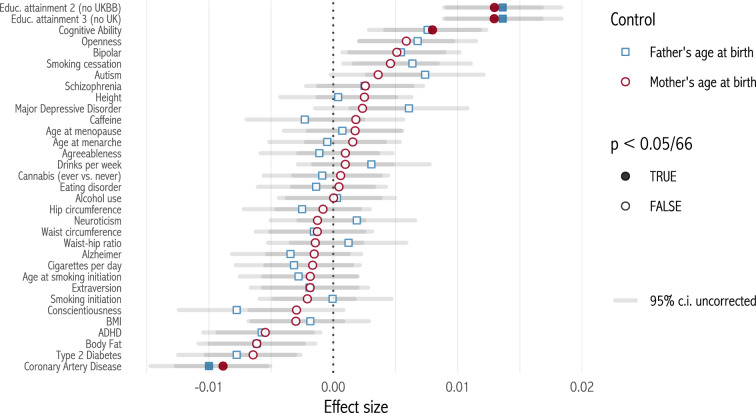
Selection effects (parents’ RLRS) among eldest siblings, controlling for parents’ age at birth. Weights multiplied by 1/number of siblings

**Fig. 17:**
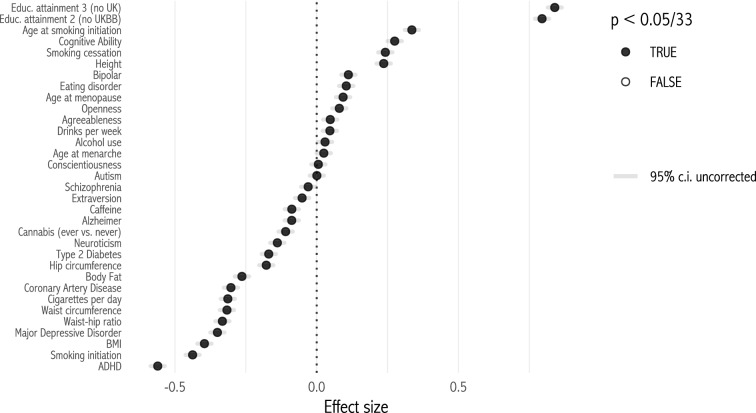
Effects of polygenic scores on age at first live birth

**Fig. 18:**
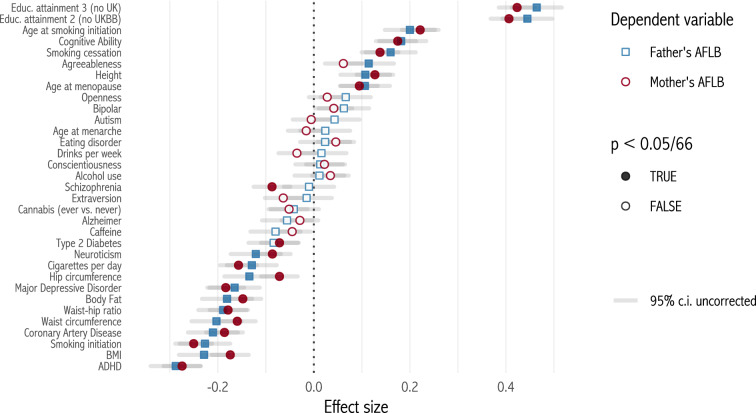
Effects of polygenic scores on parents’ age at respondent’s birth, eldest siblings. Weights multiplied by 1/number of siblings

**Fig. 19:**
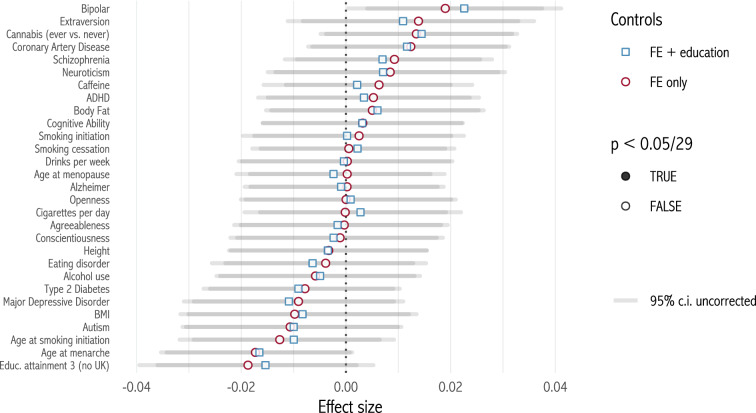
Selection effects controlling for sibling-group fixed effects, with and without a control for education (left education before 16, 16-18, or after 18). Each set of 29 results is from a single regression of RLRS on 29 polygenic scores. Standard errors clustered by sibling group

**Fig. 20:**
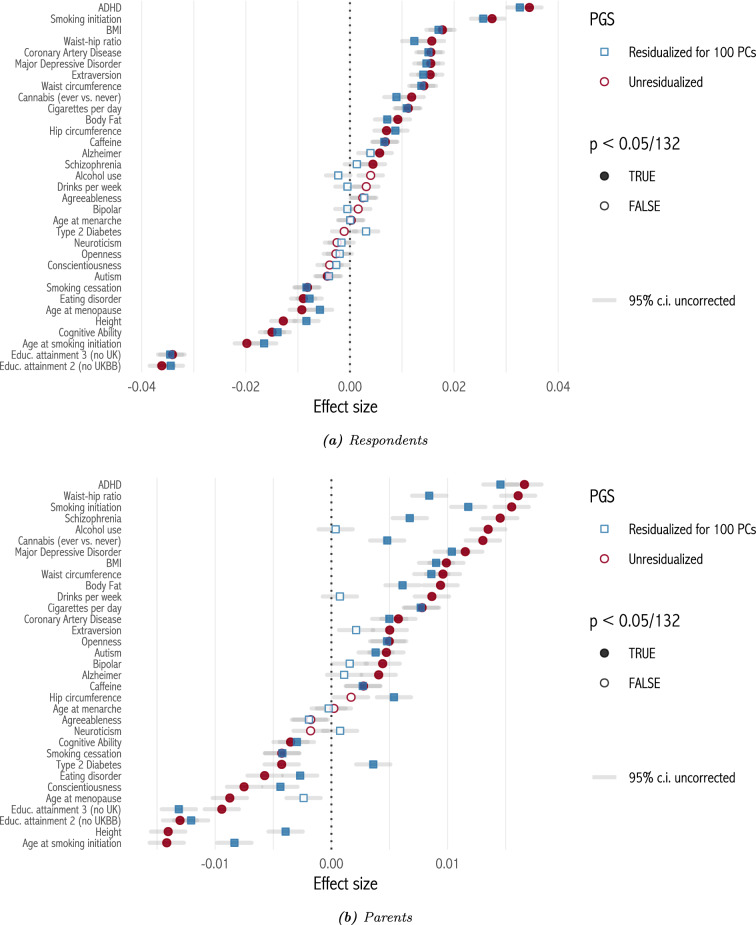
Selection effects using unresidualized polygenic scores. Parental generation weights multiplied by 1/number of siblings

**Fig. 21:**
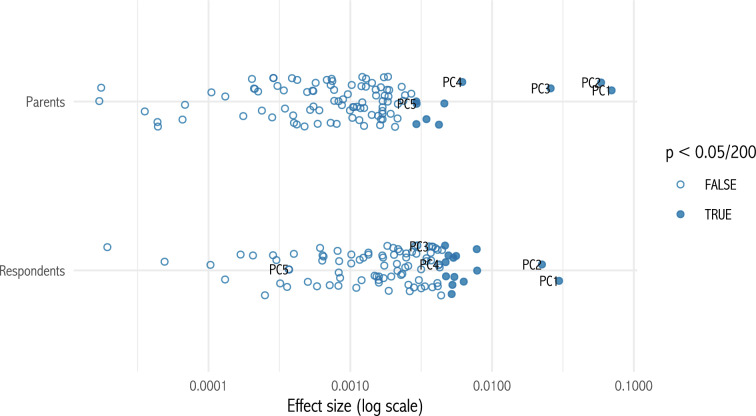
Selection effects of 100 principal components of genetic data. Each dot represents one bivariate regression. Parental generation weights multiplied by 1/number of siblings. Absolute effect sizes are plotted. Points are jittered on the Y axis. Top principal components are labelled

**Fig. 22:**
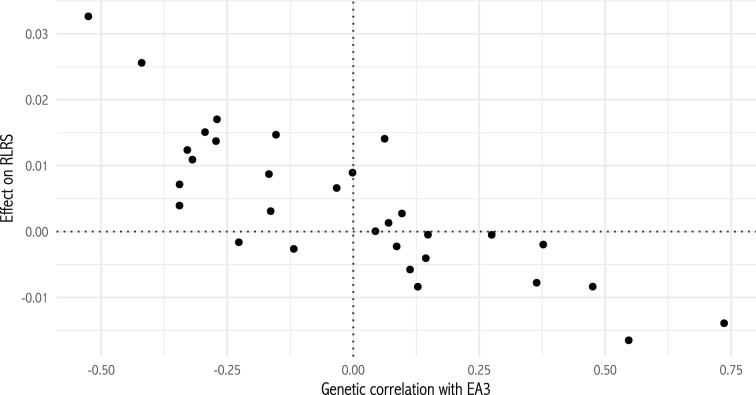
Selection effects plotted against genetic correlation with EA3

**Fig. 23:**
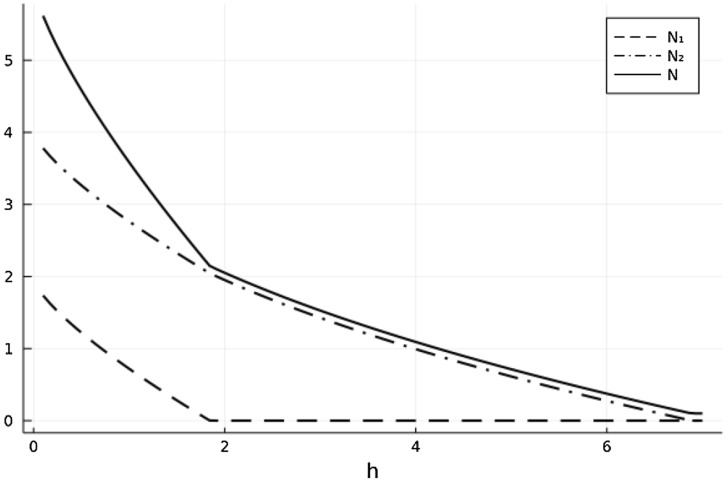
Fertility vs. human capital in the two-period model with $a=0.4,b=0.25,\sigma =0.7$

**Fig. 24:**
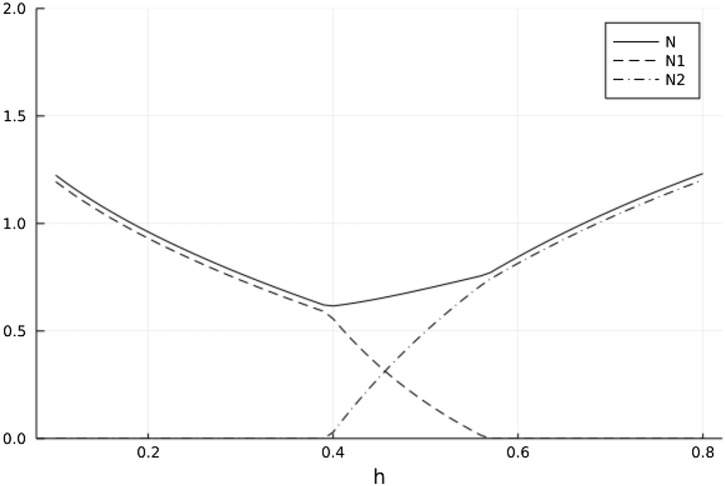
Fertility vs. human capital in the two-period model with money costs of children. $a=0.4,b=0.175,\sigma =0.7, m = 0.075$

## Data Availability

Code for this paper is available at https://github.com/hughjonesd/why-natural-selection.

## Appendix

### Selection Effects by Sex

Figure 9 plots selection effects by sex. Differences are particularly large for educational attainment, height, ADHD and MDD. Several polygenic scores for mental illness and personality traits are more selected for (or less against) among women, including major depressive disorder (MDD), schizophrenia and neuroticism, while extraversion is more selected for among men.

### Alternative Weighting Schemes

We compare results for our main weights to 3 alternative weighting schemes: weighting by age/qualification; geographical (weighting by Middle Super Output Area); and for women only, age, qualification and age at first live birth. Population data for weighting is taken from the 2011 UK Census and the 2006 General Household Survey (GHS). Weighting for Age/Qualification and Age/Qualification/AFLB weights was done using marginal totals from a linear model, using the calibrate() function in the R “survey” package (Jones and Tertilt 2020). Geographical weighting was done with iterative post-stratification using the rake() function, on Census Middle Layer Super Output Areas, sex and presence/absence of a partner.

Table 2 gives effect sizes as a proportion of the unweighted effect size, for all polygenic scores which are consistently signed and which are significantly different from zero in unweighted regressions.

**Table 2:** Weighted effect sizes as a proportion of unweighted effect sizes

Weighting				
PGS	Main	Geographical	Age/Qualification	Age/Qual/AFLB
Eating disorder	2.19	1.62	1.69	0.76
Waist-hip ratio	1.89	1.54	1.19	0.96
Coronary Artery Disease	1.79	0.97	1.17	1.09
Height	1.76	1.85	1.31	0.98
Educ. attainment 2 (no UKBB)	1.63	1.51	1.25	1.14
Educ. attainment 3 (no UK)	1.62	1.57	1.24	1.17
Major Depressive Disorder	1.61	1.44	1.24	1.01
Age at smoking initiation	1.59	1.38	1.13	0.97
Waist circumference	1.58	1.52	1.09	1.19
ADHD	1.54	1.14	1.15	1.03
Age at menopause	1.54	1.63	1.17	0.85
Cigarettes per day	1.54	1.73	0.97	0.98
Smoking initiation	1.53	1.30	1.07	0.87
BMI	1.52	1.68	1.01	1.20
Caffeine	1.48	0.86	1.14	1.44
Hip circumference	1.44	1.51	0.98	1.44
Cannabis (ever vs. never)	1.38	1.41	1.02	0.83
Cognitive Ability	1.25	1.21	1.09	1.59
Body Fat	1.23	1.36	1.13	1.16
Extraversion	1.13	0.91	1.08	2.47
Autism	0.81	1.51	0.57	−0.70
Agreeableness	0.45	0.99	0.63	13.30
Mean	1.48	1.39	1.11	1.62
Median	1.54	1.47	1.13	1.06

Only consistently-signed and significant (when unweighted) estimates are shown. Age/Qual/AFLB as a proportion of unweighted regressions including females only

### Stabillizing and Disruptive Selection

Stabilizing selection reduces variance in the trait under selection, while disruptive selection increases variance. To check for these, we rerun Eq. (1), adding a quadratic term in $PGS_i$. Scores for hip circumference show significant stabilizing selection ($p < 0.05/33$, negative coefficient on quadratic term). The EA2 score for educational attainment shows significant disruptive selection ($p < 0.05/33$, positive coefficient), which reduces the strength of selection against educational attainment at very high levels of the PGS. (The quadratic on the EA3 score has a similar coefficient but is not significant at $p < 0.05/33$.) Figure 10 plots predicted number of children against polygenic score from these regressions.

We also checked for stabilizing selection in the parents’ generation, using weights multiplied by the inverse of number of siblings. Scores for EA2 and EA3 show significant disruptive selection ($p < 0.05/33$, positive coefficient on quadratic). Other scores including hip circumference were not significant.

### Controlling for Age

Results in Fig. 3 could be explained by age, if older respondents have lower income and are less educated, and also show more natural selection on polygenic scores. However, when we rerun the regressions, interacting the polygenic score with income category and also with a quadratic in age, the interaction with income remains significant at *p* < 0.05/33 for 17 out of 33 regressions. Similarly if we interact the PGS with age of leaving full time education and a quadratic in age, the interaction with age leaving full time education remains significant at *p* < 0.05/33 for 12 out of 33 regressions.

### Number of Partners and Presence of Partner by Sex

Figure 11 splits up Fig. 4 by sex. The pattern of results is the same in both sexes: selection effects are stronger among those with more lifetime sexual partners, and among those not currently living with a partner.

### Parents’ Generation

#### Selection Effects and Change Over Time

The UK Biobank data contains information on respondents’ number of siblings (including them), i.e. their parents’ number of children. Since respondents’ polygenic scores are equal in expectation to the mean scores of their parents, we can use this to look at selection effects in the parents’ generation. We estimate equation (1) using parents’ RLRS as the dependent variable.^7^ The parents’ generation has an additional source of ascertainment bias: sampling parents of respondents overweights parents who have many children. For instance, parents of three children will have, on average, three times more children represented in UK Biobank than parents of one child. Parents of no children will by definition not be represented. To compensate, we multiply our weights by the inverse of *number of siblings*.

Figure 12 shows regressions of parents’ RLRS on polygenic scores. For a clean comparison with the respondents’ generation, we rerun regressions on respondents’ RLRS excluding those with no children, and show results in the figure. Selection effects are highly correlated across the two generations, and most share the same sign. Absolute effect size estimates are larger for the parents’ generation. We treat this result cautiously, because effect sizes in both generations may depend on polygenic scores’ correlation with childlessness, and we cannot estimate this for the parents’ generation.

To learn more about this, we compare effect sizes excluding and including childless people in the *current* generation. The correlation between the two sets of effect sizes is 0.95. So, patterns across different scores are broadly similar whether the childless are counted or not. However, absolute effect sizes are smaller when the childless are excluded, for 27 out of 33 scores; the median percentage change is –41%.

The fact that childless people have such a strong effect on estimates makes it hard to compare total effect sizes across generations. In particular, since the parents’ generation has a different distribution of numbers of children, childless people may have had more or less effect in that generation. Another issue is that we are estimating parents’ polygenic scores by the scores of their children. This introduces noise into our independent variable, which might lead to errors-in-variables and bias coefficients towards zero.

As an alternative approach, we run regressions interacting polygenic scores with birth year, median split at 1950 (“early born” versus “late born”). We use both respondents’ RLRS and parents’ RLRS as a dependent variable. We use our standard weights, and further adjust for selection in the parents’ generation (see above).

Table 3 summarizes the results. We report the number of scores showing significant changes over time (i.e. a significant interaction between polygenic score and the “late born” dummy): either a significant change in sign, a significant increase in effect size, or a significant decrease in size. There is little evidence for changes in selection effects within the parents’ generation, with just one score showing a significant decrease in size. In the respondents’ generation, effect sizes were significantly larger in absolute size among the later-born for eight polygenic scores: ADHD, age at menopause, cognitive ability, Coronary Artery Disease, EA2, EA3, extraversion and Major Depressive Disorder. These changes are inconsistent with the intergenerational change, where estimated effect sizes were larger among the earlier, parents’ generation.

Overall, while there is some suggestive evidence for an increase in the strength of selection in recent history, the clearest result is that the pattern of relative effect sizes across scores is broadly consistent over time.

**Table 3:** Numbers of polygenic scores showing changes in selection effects between early and late born. Parental generation weights multiplied by 1/number of siblings

Change	Parents’ RLRS	Respondents’ RLRS
Insignificant	32	25
Size decreasing	1	
Size increasing		8

Significance is measured at p < 0.05/66

#### Area Deprivation

Figure 13 plots effects on parents’ RLRS by Townsend deprivation quintile of birth area.

For comparison, Fig. 14 plots effects on respondents’ RLRS by Townsend deprivation quintile of birth area.

#### Age at First Live Birth

Among the parents’ generation, we can control for age at first live birth using the subsets of respondents who reported their mother’s or father’s age, and who had no elder siblings. We run regressions on parents’ RLRS on these subsets. Figure 15 shows selection effects by terciles of age at first live birth, for mothers and fathers. As in the respondents’ generation, effect sizes are smaller, or even oppositely signed, for older parents. Importantly, this holds for both sexes.

Figure 16 shows the regressions controlling for either parent’s age at their birth. Effect sizes are very similar, whether controlling for father’s or mother’s age. As in the respondents’ generation, effect sizes are negatively correlated with the effect sizes from bivariate regressions without the control for age at birth (father’s age at birth: $\rho$ –0.43; mother’s age at birth: $\rho$ –0.59).

### Effects of Polygenic Scores on Age at First Live Birth

Our results suggest that polygenic scores may directly correlate with age at first live birth. Figure 17 plots estimated effect sizes from bivariate regressions for respondents. Figure 18 does the same for their parents, using only eldest siblings.^8^ Effect sizes are reasonably large. They are also highly correlated across generations. Effect sizes of polygenic scores on father’s age at own birth, and on own age at first live birth, have a correlation of 0.99; for mother’s age and own age it is 0.99.

### Mediation Analysis

We run a standard mediation analysis in the framework of Baron and Kenny (1986). For each polygenic score where the bivariate correlation with RLRS is significant at *p* < 0.05/33, we estimate

3 $$\begin{aligned} RLRS_i&= \alpha + \beta PGS_i + \gamma EA_i + X_i\mu + \varepsilon _i \end{aligned}$$

4 $$\begin{aligned} EA_i&= \delta + \zeta PGS_i + X_i\mu + \eta _i \end{aligned}$$

where $RLRS_i$ is relative lifetime reproductive success, $PGS_i$ is the polygenic score, $EA_i$ is educational attainment (age of leaving fulltime education), and $X_i$ is a vector of controls. The total effect of *PGS* on *RLRS* is $\beta + \gamma \zeta$. The “indirect effect” mediated by *EA* is $\gamma \zeta$. The standard error of the indirect effect can be calculated as

$$\begin{aligned} \sqrt{{\hat{\gamma }}^2 {\hat{\sigma }}_\zeta ^2 + {\hat{\zeta }}^2{\hat{\sigma }}_\gamma ^2} \end{aligned}$$

where ${\hat{\sigma }}_\zeta$ is the standard error of ${\hat{\zeta }}$, etc. We include controls for age and sex in *X*.

**Table 4:** Mediation analysis

PGS	Total effect	Indirect effect	Proportion (%)	Proportion 95% c.i. (%)
ADHD	0.0262	0.0054 *	20.7	[16.5, 28.1]
Smoking initiation	0.0229	0.0046 *	20.2	[16.2, 28.5]
BMI	0.0181	0.0034 *	19.1	[14.1, 29.2]
Major Depressive Disorder	0.0146	0.0017 *	11.5	[8.1, 18.0]
Waist circumference	0.0144	0.0033 *	22.9	[16.2, 37.1]
Extraversion	0.0127	0.0006 *	4.9	[1.9, 10.6]
Hip circumference	0.0117	0.0018 *	15.5	[10.0, 33.5]
Waist-hip ratio	0.0107	0.0035 *	32.9	[21.4, 56.5]
Coronary Artery Disease	0.0106	0.0033 *	30.8	[21.1, 54.8]
Cigarettes per day	0.0088	0.0026 *	30.2	[18.4, 129.0]
Body Fat	0.0072	0.0029 *	40.8	[21.7, 120.6]
Caffeine	0.0054	0.0000	0.5	Unbounded
Cannabis (ever vs. never)	0.0049	0.0006 *	11.8	Unbounded
Alzheimer	0.0049	0.0013 *	26.6	Unbounded
Age at menopause	−0.0048	−0.0012 *	25.2	Unbounded
Autism	−0.0048	−0.0015 *	31.2	Unbounded
Eating disorder	−0.0081	−0.0019 *	23.9	[13.7, 73.5]
Height	−0.0087	−0.0020 *	23.2	[13.1, 84.1]
Smoking cessation	−0.0092	−0.0028 *	30.7	[17.7, 71.5]
Cognitive Ability	−0.0138	−0.0054 *	39.2	[29.1, 64.3]
Age at smoking initiation	−0.0153	−0.0033 *	21.3	[15.7, 33.1]
Educ. attainment 3 (no UK)	−0.0302	−0.0114 *	37.8	[30.2, 46.8]
Educ. attainment 2 (no UKBB)	−0.0305	−0.0113 *	36.9	[29.5, 46.5]

* p < 0.05/23. Analysis run on 23 PGS which correlated significantly with fertility

Table 4 shows results. For 22 out of 23 scores, the indirect effect on fertility via human capital is significantly different from 0 at *p* = 0.05/23 and has the same sign as the total effect. We also calculate the proportion of the total effect that is mediated via the indirect effect, along with uncorrected 95% confidence intervals (100 bootstraps). Note that if the confidence interval for the total effect contains zero, the confidence interval for the proportion may be unbounded (Franz 2007).

### Within-Siblings Regressions

Results in the main text support our theory that natural selection on polygenic scores is driven by their *correlation* with human capital. Here, we test whether polygenic scores *cause* fertility by running within-siblings regressions. We run a single regression on 29 polygenic scores within 17161 sibling groups (N = 31169). Thus, we control both for environmental confounds (since scores are randomly allocated within sib-groups by meiosis), and for genetic confounds captured by our polygenic scores. We remove four scores which correlate highly with other scores (educational attainment 2, hip circumference, waist circumference and waist-hip ratio). Figure 19 shows the results.

With a reduced sample size, all within-sibling effects are insignificant after Bonferroni correction. However, effect sizes are positively correlated with effect sizes from the pooled model, and about 70% smaller (regressing within-sibling on pooled effect sizes, *b* = 0.292). This attenuation is broadly consistent with the decrease in heritability in within-sibling GWASs on age at first birth and educational attainment (Howe et al. 2021). We see these results as providing tentative evidence that polygenic scores cause fertility, with effects being partly driven by correlations with environmental variation in human capital. We also reran within-siblings regressions adding a control for education. Most effect sizes barely change, suggesting that our measure of education does not in general mediate differences in fertility among siblings.

### Effects on Inequality

Table 5 shows correlations between children’s polygenic scores and household income (UKB data field 738). Column “With selection” uses respondents’ scores, multiplying weights by number of children. Column “Without selection” uses our standard weights, i.e.  it estimates the counterfactual correlation if all respondents had the same number of children.

**Table 5:** Correlations of polygenic scores with income group

PGS	Cor. with selection	Cor. without selection	Ratio
Educ. attainment 3 (no UK)	0.163	0.141	1.15
Educ. attainment 2 (no UKBB)	0.155	0.135	1.15
Cognitive Ability	0.058	0.053	1.08
Age at smoking initiation	0.049	0.039	1.26
Smoking cessation	0.047	0.042	1.13
Height	0.043	0.039	1.09
Eating disorder	0.020	0.018	1.10
Agreeableness	0.018	0.017	1.02
Openness	0.016	0.014	1.19
Extraversion	0.014	0.016	0.92
Age at menopause	0.014	0.012	1.18
Bipolar	0.014	0.012	1.17
Drinks per week	0.009	0.009	1.08
Alcohol use	0.005	0.006	0.83
Age at menarche	0.004	0.002	1.82
Conscientiousness	0.003	0.001	3.63
Autism	−0.009	−0.009	1.00
Caffeine	−0.011	−0.011	1.00
Alzheimer	−0.012	−0.011	1.06
Cannabis (ever vs. never)	−0.015	−0.008	1.72
Schizophrenia	−0.027	−0.029	0.94
Type 2 Diabetes	−0.030	−0.025	1.24
Neuroticism	−0.031	−0.031	1.02
Hip circumference	−0.033	−0.027	1.24
Body Fat	−0.050	−0.043	1.15
Cigarettes per day	−0.052	−0.043	1.22
Coronary Artery Disease	−0.053	−0.039	1.33
Waist circumference	−0.057	−0.048	1.18
Waist-hip ratio	−0.060	−0.051	1.17
Major Depressive Disorder	−0.063	−0.054	1.16
BMI	−0.065	−0.052	1.24
Smoking initiation	−0.074	−0.059	1.25
ADHD	−0.095	−0.077	1.24

### Further Results

#### Selection Effects on Raw Polygenic Scores

Figure 20 compares selection effects on polygenic scores residualized for the top 100 principal components of the genetic data, to selection effects on raw, unresidualized polygenic scores. In siblings regressions, effect sizes are larger for raw scores—sometimes much larger, as in the case of height. 29 out of 33 “raw” effect sizes have a larger absolute value than the corresponding “residualized” effect size. The median proportion between raw and controlled effect sizes is 0.8. Among the children regressions, this no longer holds. Effect sizes are barely affected by controlling for principal components.

Overall, 72.73 per cent of effect sizes are consistently signed across all four regressions (on children and siblings, and with and without residualization).

To get a further insight into this we regress respondents’ and parents’ RLRS on individual principal components. Figure 21 shows the results. Labels show the top principal components. These have larger effect sizes in siblings regressions. One possibility is that the parents’ generation was less geographically mobile, and so geographic patterns of childrearing were more correlated with principal components, which partly capture the location of people’s ancestors.

#### Genetic Correlations with EA3

Another way to examine the “earnings” theory of natural selection is to compare selection effects of polygenic scores with their genetic correlation with educational attainment (EA3). Since EA3 strongly predicts earnings, if earnings drives differences in fertility, we’d expect a correlation between the two sets of results. Figure 22 shows this is so: the correlation, after excluding EA2, is –0.82. Genetic correlations were calculated using LD score regression from GWAS summary statistics.

## Solution for the One-Period Model

Differentiating and setting $\frac{dU}{dN}=0$ gives the first order condition for an optimal choice of children $N^{*}>0$:

$$\begin{aligned} \frac{bW}{(W(1-bN^{*}))^{\sigma }}\ge a\text {, with equality if }N^{*}>0. \end{aligned}$$

Rearranging gives

5 $$\begin{aligned} N^{*}=\max \left\{ \frac{1}{b}\left( 1-\left( \frac{b}{a}\right) ^{1/\sigma }W^{(1-\sigma )/\sigma }\right) ,0\right\} . \end{aligned}$$

Note that when $\sigma <1$, for high enough *W*, $N^{*}=0$. Differentiating gives the effect of wages on fertility for $N^{*}>0$. This is also the fertility-human capital relationship:

6 $$\begin{aligned} \frac{dN^{*}}{dh}=\frac{dN^{*}}{dW}=-\frac{1}{b}\left( \frac{b}{a}\right) ^{1/\sigma }\frac{1-\sigma }{\sigma }W^{(1-2\sigma )/\sigma }. \end{aligned}$$

This is negative if $\sigma <1$. Also,

$$\begin{aligned} \frac{d^{2}N^{*}}{dW^{2}}=-\frac{1}{b}\left( \frac{b}{a}\right) ^{1/\sigma }\frac{1-\sigma }{\sigma }\frac{1-2\sigma }{\sigma }W^{(1-3\sigma )/\sigma } \end{aligned}$$

For $0.5<\sigma <1$, this is positive, so the effect of fertility on wages shrinks towards zero as wages increase (and becomes 0 when $N^{*}=0$). Next, we consider the time cost of children *b*:

$$\begin{aligned} \frac{d^{2}N^{*}}{dWdb}=-\left( \frac{1}{a}\right) ^{1/\sigma }\left( \frac{1-\sigma }{\sigma }\right) ^{2}(Wb)^{(1-2\sigma )/\sigma }<0. \end{aligned}$$

Lastly we consider the effect of *a*. From (5), $N^{*}$ is increasing in *a*. Differentiating (6) by *a* gives

$$\begin{aligned} \frac{d^{2}N^{*}}{dadW}=b^{1/\sigma -1}\frac{1-\sigma }{\sigma ^{2}}W^{(1-2\sigma )/\sigma }a^{-1/\sigma -1} \end{aligned}$$

which is positive for $\sigma <1$.

## Solution for the Two-Period Model

Period 1 and period 2 income are:

7 $$\begin{aligned} Y_{1}&=1-s-bN_{1} \end{aligned}$$

8 $$\begin{aligned} Y_{2}&=w(s,h)(1-bN_{2}) \end{aligned}$$

Write the Lagrangian of utility *U* (2) as

$$\begin{aligned} {\mathcal {L}}(N_{1},N_{2},s)=u(Y_{1})+u(Y_{2})+a(N_{1}+N_{2})+\lambda _{1}N_{1}+\lambda _{2}N_{2}+\lambda _{3}(\frac{1}{b}-N_{2})+\mu s \end{aligned}$$

Lemma 5 below shows that if $\sigma >0.5$, this problem is globally concave, guaranteeing that the first order conditions identify a unique solution. We assume $\sigma >0.5$ from here on.

Plugging (7) and (8) into the above, we can derive the Karush-Kuhn-Tucker conditions for an optimum $(N_{1}^{*},N_{2}^{*},s^{*})$ as:

9 $$\begin{aligned}&\frac{d{\mathcal {L}}}{dN_{1}}=-bY_{1}^{-\sigma }+a+\lambda _{1} =0\text {, with }\lambda _{1}=0\text { if }N_{1}^{*}>0; \end{aligned}$$

10 $$\begin{aligned}&\frac{d{\mathcal {L}}}{dN_{2}}=-bs^{*}hY_{2}^{-\sigma }+a+\lambda _{2} -\lambda _{3}=0\text {, with }\lambda _{2}=0\text { if }N_{2}^{*}>0,\lambda _{3}=0\text { if }N_{2}^{*}<\frac{1}{b}; \end{aligned}$$

11 $$\begin{aligned}&\frac{d{\mathcal {L}}}{ds}=-Y_{1}^{-\sigma }+h(1-bN_{2}^{*})Y_{2}^{-\sigma }+\mu =0; \end{aligned}$$

12 $$\begin{aligned}&N_{1}^{*},N_{2}^{*},s^{*},\lambda _{1},\lambda _{2},\lambda _{3},\mu \ge 0;N_{2}^{*}\le \frac{1}{b}. \end{aligned}$$

Note that the Inada condition (that marginal utility of income grows without bound as income approaches zero, $\lim _{x\rightarrow 0}u'(x)=\infty$) for period 1 rules out $s^{*}=1$ and $N_{1}=1/b$, so we need not impose these constraints explicitly. Also, so long as $N_{2}^{*}<1/b$, the same condition rules out $s^{*}=0$. We consider four cases, of which only three can occur.

### Case 1: $N_{1}^{*}>0,N_{2}^{*}>0$

Rearranging (9), (10) and (11) gives:

13 $$\begin{aligned} N_{1}^{*}&=\frac{1}{b}\left( 1-s^{*}-\left( \frac{b}{a}\right) ^{1/\sigma }\right) ; \end{aligned}$$

14 $$\begin{aligned} N_{2}^{*}&=\frac{1}{b}\left( 1-\left( \frac{b}{a}\right) ^{1/\sigma }(s^{*}h)^{(1-\sigma )/\sigma }\right) ; \end{aligned}$$

15 $$\begin{aligned} s^{*}&=\frac{1-bN_{1}^{*}}{1+\left( (1-bN_{2}^{*})h\right) ^{1-1/\sigma }}. \end{aligned}$$

Plugging the expressions for $N_{1}^{*}$ and $N_{2}^{*}$ into $s^{*}$ gives

$$\begin{aligned} s^{*}=\frac{s^{*}+\left( \frac{b}{a}\right) ^{1/\sigma }}{1+\left( \left( \frac{b}{a}\right) ^{1/\sigma }s^{*(1-\sigma )/\sigma }h{}^{1/\sigma }\right) ^{1-1/\sigma }} \end{aligned}$$

which simplifies to

16 $$\begin{aligned} s^{*}=\left( \frac{b}{a}\right) ^{1/(2\sigma -1)}h^{(1-\sigma )/(2\sigma -1)}. \end{aligned}$$

Plugging the above into (13) and (14) gives:

$$\begin{aligned} N_{1}^{*}&=\frac{1}{b}\left( 1-\left( \frac{b}{a}\right) ^{1/(2\sigma -1)}h^{(1-\sigma )/(2\sigma -1)}-\left( \frac{b}{a}\right) ^{1/\sigma }\right) ;\\ N_{2}^{*}&=\frac{1}{b}\left( 1-\left( \frac{b}{a}\right) ^{1/(2\sigma -1)}h^{(1-\sigma )/(2\sigma -1)}\right) . \end{aligned}$$

Note that that $N_{1}^{*}<N_{2}^{*}$. For these both to be positive requires low values of *h* if $\sigma <1$ and high values of *h* if $\sigma >1$. Also:

$$\begin{aligned} w(s^{*},h)\equiv s^{*}h=\left( \frac{b}{a}\right) ^{1/(2\sigma -1)}h^{\sigma /(2\sigma -1)}. \end{aligned}$$

Observe that $w(s^{*},h)$ is increasing in *h* for $\sigma >0.5$, and convex iff $0.5<\sigma <1$.

While $N_{1}^{*}$ and $N_{2}^{*}$ are positive, they have the same derivative with respect to *h*:

17 $$\begin{aligned} \frac{dN_{t}^{*}}{dh}=-\frac{1}{b}\left( \frac{b}{a}\right) ^{1/(2\sigma -1)}\frac{1-\sigma }{2\sigma -1}h^{(1-\sigma )/(2\sigma -1)-1} \end{aligned}$$

Examining this and expression (16) gives:

#### Lemma 1

For $\sigma <1$, case 1 holds for *h* low enough, and in case 1, $N_{1}^{*}$ and $N_{2}^{*}$ decrease in *h*, while $s^{*}$ increases in *h*.

For $\sigma >1$, case 1 holds for *h* high enough, and in case 1 $N_{1}^{*}$ and $N_{2}^{*}$ increase in *h*, while $s^{*}$ decreases in *h*.

$N_{t}^{*}$ is convex in *h* for $\sigma >2/3$, and concave otherwise. $s^{*}$ is convex in *h* if $\sigma <2/3$, and concave otherwise.

### Case 2: $N_{1}^{*}=0,N_{2}^{*}>0$

Replace $N_{1}^{*}=0$ into the first order condition for $s^{*}$ from (11), and rearrange to give:

$$\begin{aligned} s^{*}=\frac{1}{1+\left( (1-bN_{2})h\right) ^{1-1/\sigma }}. \end{aligned}$$

Now since $N_{2}^{*}>0$, we can rearrange (10) to give

18 $$\begin{aligned} N_{2}^{*}=\frac{1}{b}\left( 1-\left( \frac{b}{a}\right) ^{1/\sigma }(s^{*}h)^{(1-\sigma )/\sigma }\right) . \end{aligned}$$

Plugging this into $s^{*}$ gives

$$\begin{aligned} s^{*}&=\frac{1}{1+\left( \frac{bh}{a}\right) ^{(\sigma -1)/\sigma ^{2}}(s^{*}){}^{-(1-\sigma )^{2}/\sigma ^{2}}} \end{aligned}$$

which can be rearranged to

19 $$\begin{aligned} (1-s^{*})(s^{*})^{(1-2\sigma )/\sigma ^{2}}=\left( \frac{a}{bh}\right) ^{(1-\sigma )/\sigma ^{2}}. \end{aligned}$$

Differentiate the left hand side of the above to get

20 $$\begin{aligned}&\frac{1-2\sigma }{\sigma ^{2}}(1-s^{*})(s^{*})^{(1-2\sigma )/\sigma ^{2}-1}-(s^{*})^{(1-2\sigma )/\sigma ^{2}}\nonumber \\&\quad = \frac{1-2\sigma }{\sigma ^{2}}(s^{*})^{(1-2\sigma )/\sigma ^{2}-1}-\frac{\sigma ^{2}+1-2\sigma }{\sigma ^{2}}(s^{*})^{(1-2\sigma )/\sigma ^{2}}\nonumber \\&\quad = \frac{1-2\sigma }{\sigma ^{2}}(s^{*})^{(1-2\sigma )/\sigma ^{2}-1}-\frac{(1-\sigma )^{2}}{\sigma ^{2}}(s^{*})^{(1-2\sigma )/\sigma ^{2}}. \end{aligned}$$

This is negative if and only if

$$\begin{aligned} s^{*}&>\frac{1-2\sigma }{(1-\sigma )^{2}} \end{aligned}$$

which is always true since $\sigma >0.5$. Note also that since $\sigma >0.5$, then the left hand side of (19) approaches infinity as $s^{*}\rightarrow 0$ and approaches 0 as $s^{*}\rightarrow 1$. Thus, (19) implicitly defines the unique solution for $s^{*}$.

To find how $s^{*}$ changes with *h*, note that the right hand side of the above decreases in *h* for $\sigma <1$, and increases in *h* for $\sigma >1$. Putting these facts together: for $\sigma <1$, when *h* increases the RHS of (19) decreases, hence the LHS decreases and $s^{*}$ increases, i.e. $s^{*}$ is increasing in *h*. For $\sigma >1$, $s^{*}$ is decreasing in *h*.

To find how $N_{2}^{*}$ changes with *h*, we differentiate (18):

21 $$\begin{aligned} \frac{dN_{2}^{*}}{dh}&=-\frac{1}{b}\left( \frac{b}{a}\right) ^{1/\sigma }\frac{1-\sigma }{\sigma }(s^{*}h)^{(1-2\sigma )/\sigma }(s^{*}+h\frac{ds^{*}}{dh}) \end{aligned}$$

which is negative for $\sigma <1$, since $\frac{ds^{*}}{dh}>0$ in this case.

Differentiating again:

$$\begin{aligned} \frac{d^{2}N_{2}}{dh^{2}}&=-X \left[ \frac{1-2\sigma }{\sigma }(s^{*}h)^{(1-3\sigma )/\sigma }(s^{*}+h\frac{ds^{*}}{dh})^{2}\right. \\&\quad \left. +(s^{*}h)^{(1-2\sigma )/\sigma }(2\frac{ds^{*}}{dh}+h\frac{d^{2}s^{*}}{dh^{2}})\right] \\&=X(s^{*}h)^{(1-3\sigma )/\sigma }\left[ \frac{2\sigma -1}{\sigma }(s^{*}\right. \\&\left. \quad +h\frac{ds^{*}}{dh})^{2}-(s^{*}h)(2\frac{ds^{*}}{dh}+h\frac{d^{2}s^{*}}{dh^{2}})\right] \end{aligned}$$

where $X=\frac{1}{b}\left( \frac{b}{a}\right) ^{1/\sigma }\frac{1-\sigma }{\sigma }>0$. Note that $\frac{d^{2}N_{2}}{dh^{2}}$ is continuous in $\sigma$ around $\sigma =1$. Note also from (19) that for $\sigma =1$, $s^{*}$ becomes constant in $\sigma$. The term in square brackets then reduces to $(s^{*})^{2}>0$. Putting these facts together, for $\sigma$ sufficiently close to 1, $\frac{d^{2}N_{2}^{*}}{dh^{2}}>0$, i.e. $N_{2}^{*}$ is convex in *h*.

This case holds for intermediate values on *h*. Equation (21) shows that for $\sigma < 1$, $N_2$ decreases in *h*; the requirement that $N_2>0$ therefore puts a maximum on *h*. When $\sigma >1$, $N_2$ increases in *h* and this puts a minimum on *h*. The requirement $N_1 = 0$ provides the other bound. Equation (9) requires $-bY_{1}^{-\sigma }+a \le 0$ since $\lambda _1$ must be non-negative. The LHS is increasing in $Y_1$, and hence decreasing in *s* as $Y_1 = 1-s$ since $N_1=0$. Lastly, optimal choice of education $s^*$ increases in *h* for $\sigma <1$, and decreases for $\sigma > 1$. Hence for $\sigma < 1$, (9) puts a minimum on *h*, and for $\sigma > 1$ it puts a maximum on *h*.

Summarizing:

#### Lemma 2

Case 2 holds for intermediate values of *h*. In case 2: for $\sigma <1$, $s^{*}$ is increasing in *h* and $N_{2}^{*}$ is decreasing in *h*. For $\sigma >1$, $s^{*}$is decreasing in *h*. For $\sigma$ close enough to 1, $N_{2}^{*}$ is convex in *h*.

### Case 3: $N_{1}^{*}=0,N_{2}^{*}=0$

We can solve for $s^{*}$ by substituting values of $Y_{1}$ and $Y_{2}$ into (11):

$$\begin{aligned} -(1-s^{*}){}^{-\sigma }+h(s^{*}h)^{-\sigma }=0 \end{aligned}$$

which rearranges to

22 $$\begin{aligned} s^{*}=\frac{1}{1+h^{(\sigma -1)/\sigma }}. \end{aligned}$$

Conditions (9) and (10) become:

$$\begin{aligned} -b(1-s^{*})^{-\sigma }+a&\le 0\\ -bs^{*}h(s^{*}h)^{-\sigma }+a&\le 0 \end{aligned}$$

equivalently

$$\begin{aligned} \frac{a}{b}&\le (1-s^{*})^{-\sigma }\\ \frac{a}{b}&\le s^{*}h(s^{*}h)^{-\sigma } \end{aligned}$$

which can both be satisfied for *a*/*b* close enough to zero. Note from (22) that as $h\rightarrow \infty$, $s^{*}$ increases towards 1 for $\sigma <1$, and decreases towards 0 for $\sigma >1$. Note also that the right hand side of the first inequality above approaches infinity as $s^{*}\rightarrow 1$, therefore also as $h\rightarrow \infty$ for $\sigma <1$. Rewrite the second inequality as

$$\begin{aligned} \frac{a}{b}<(s^{*}h)^{1-\sigma }=\left( \frac{h}{1+h^{(\sigma -1)/\sigma }}\right) ^{1-\sigma }=\left( h^{-1}+h^{-1/\sigma }\right) ^{\sigma -1} \end{aligned}$$

and note that again, as $h\rightarrow \infty$, the RHS increases towards infinity for $\sigma <1$, and decreases towards zero otherwise. Thus, for $\sigma <1$, both equations will be satisfied for *h* high enough. For $\sigma >1$, they will be satisfied for *h* low enough. Summarizing

#### Lemma 3

For $\sigma <1$, case 3 holds for *h* high enough, and in case 3, $s^{*}$ increases in *h*. For $\sigma >1$, case 3 holds for *h* low enough and $s^{*}$ decreases in *h*.

### Case 4: $N_{1}^{*}>0,N_{2}^{*}=0$

Rearranging the first order conditions (9) and (10) for $N_{1}^{*}$ and $N_{2}^{*}$ gives

$$\begin{aligned} \frac{a}{b}&=(1-s^{*}-bN_{1}^{*})^{-\sigma }\\ \frac{a}{b}&\le s^{*}hY_{2}^{-\sigma } \end{aligned}$$

hence

$$\begin{aligned} (1-s^{*}-bN_{1}^{*})^{-\sigma }&\le s^{*}hY_{2}^{-\sigma }=(s^{*}h)^{1-\sigma }\\ \Leftrightarrow (1-s^{*}-bN_{1}^{*})^{\sigma }&\ge (s^{*}h)^{\sigma -1}\\ \Leftrightarrow 1-s^{*}-bN_{1}^{*}&\ge (s^{*}h)^{1-1/\sigma } \end{aligned}$$

Now rearrange the first order condition for $s^{*}$ from (11), noting that since $N_{2}^{*}=0$, $s^{*}>0$ by the Inada condition.

$$\begin{aligned} h^{1/\sigma -1}(1-s^{*}-bN_{1}^{*})&=s^{*}\\ 1-s^{*}-bN_{1}^{*}&=s^{*}h^{1-1/\sigma } \end{aligned}$$

This, combined with the previous inequality, implies

$$\begin{aligned} (s^{*}h)^{1-1/\sigma }&\le s^{*}h^{1-1/\sigma }\\ \Leftrightarrow (s^{*})^{-1/\sigma }&\le 1 \end{aligned}$$

which cannot hold since $0<s^{*}<1$.

### Comparative Statics

We can now examine how the fertility-human capital relationship

$$\begin{aligned} \frac{dN^{*}}{dh},\text { where }N^{*}\equiv N_{1}^{*}+N_{2}^{*}, \end{aligned}$$

changes with respect to other parameters. We focus on the case $\sigma <1$, since it gives the closest match to our observations, and since it also generates “reasonable” predictions in other areas, e.g. that education levels increase with human capital. Figure 23 shows how $N^{*}$ changes with *h* for $a=0.4,b=0.25,\sigma =0.7$.

#### Lemma 4

For $\sigma < 1$ in a neighbourhood of 1, $N^*$ is globally convex in *h*.

#### Proof

From Lemmas 1, 2 and 3, as *h* increases we move from $N_{1}^{*},N_{2}^{*}>0$ to $N_{1}^{*}=0,N_{2}^{*}>0$ to $N_{1}^{*}=N_{2}^{*}=0$. Furthermore, for $\sigma >2/3$, $N_{1}^{*}$ and $N_{2}^{*}$ are convex in *h* when they are both positive, and for $\sigma$ close enough to 1, $N_{2}^{*}$ is convex in *h* when $N_{1}^{*}=0$. All that remains is to check that the derivative is increasing around the points where these 3 regions meet. That is trivially satisfied where $N_{2}^{*}$ becomes 0, since thereafter $\frac{dN^{*}}{dh}$ is zero. The derivative as $N_{1}^{*}$ approaches zero is twice the expression in (17):

23 $$\begin{aligned} -\frac{2}{b}\left( \frac{b}{a}\right) ^{1/(2\sigma -1)}\frac{1-\sigma }{2\sigma -1}h^{(1-\sigma )/(2\sigma -1)-1} \end{aligned}$$

and the derivative to the right of this point is given by (21):

24 $$\begin{aligned} -\frac{1}{b}\left( \frac{b}{a}\right) ^{1/\sigma }\frac{1-\sigma }{\sigma }(s^{*}h)^{(1-2\sigma )/\sigma }(s^{*}+h\frac{ds^{*}}{dh}) \end{aligned}$$

We want to prove that the former is larger in magnitude (i.e. more negative). Dividing (23) by (24) gives

$$\begin{aligned} 2\frac{\sigma }{2\sigma -1}\left( \frac{b}{a}\right) ^{(1-\sigma )/(\sigma (2\sigma -1))}\frac{h^{(1-\sigma )^{2}/(\sigma (2\sigma -1))}}{s^{*}(s^{*}+h\frac{ds^{*}}{dh})} \end{aligned}$$

Examining (19) shows that as $\sigma \rightarrow 1$, $s^{*}\rightarrow 0.5$ and $\frac{ds^{*}}{dh}\rightarrow 0$, and therefore the above approaches

$$\begin{aligned} 2\frac{1}{(0.5)^{2}}=8. \end{aligned}$$

$$\square$$

We can now gather the theoretical predictions stated in Table 1.

**Prediction 1** for $\sigma <1$, total fertility $N^{*}\equiv N_{1}^{*}+N_{2}^{*}$ is decreasing in human capital *h*.

Furthermore, for $\sigma$ close enough to 1, fertility is convex in human capital, i.e. 

**Prediction 2 part 1** the fertility-human capital relationship is closer to 0 at high levels of *h*.

For $\sigma <1$, education levels $s^{*}$ increase in *h*, and so therefore do equilibrium wages $w(s^{*},h)$. This, plus fact 1, gives:

**Prediction 2 part 2** for $\sigma <1$ and close to 1, the fertility-human capital relationship is weaker among higher earners.

**Prediction 4** for $\sigma <1$ and close to 1, the fertility-human capital relationship is weaker at high levels of education.

Next, we compare people who start fertility early ($N_{1}^{*}>0$) versus those who start fertility late ($N_{1}^{*}=0$). Again, for $\sigma <1$ the former group have lower *h* than the latter group. Thus we have:

**Prediction 5** for $\sigma <1$ and close to 1, the fertility-human capital relationship is weaker among those who start fertility late.

Lastly, we prove prediction 3. Differentiating $dN_{t}^{*}/dh$ in (17) with respect to *b*, for when $N_{1}^{*}>0$ gives:

$$\begin{aligned}&\frac{d^{2}N_{t}^{*}}{dhdb} =\frac{2\sigma -2}{2\sigma -1}b^{(3-4\sigma )/(2\sigma -1)}\\&\quad \left( \frac{1}{a}\right) ^{1/(2\sigma -1)}\frac{1-\sigma }{2\sigma -1}h^{(\sigma -1)^{2}/(\sigma (2\sigma -1))} \end{aligned}$$

which is negative for $0.5<\sigma <1$. When $N_{1}^{*}=0$, differentiating $dN_{2}^{*}/dh$ in (21) gives:

$$\begin{aligned}&\frac{d^{2}N_{2}^{*}}{dhdb}=-\frac{1-\sigma }{\sigma }b^{(1-2\sigma )/\sigma }\\&\quad \left( \frac{1}{a}\right) ^{1/\sigma }\frac{1-\sigma }{\sigma }(s^{*}h)^{(1-2\sigma )/\sigma }(s^{*}+h\frac{ds^{*}}{dh}) \end{aligned}$$

which again is negative for $\sigma <1$. Therefore:

**Prediction 3** for $\sigma <1$, the fertility-human capital relationship is more negative when the burden of childcare *b* is larger.

### Including a Money Cost

The model can be extended by adding a money cost *m* per child. Utility is then

$$\begin{aligned} U = u(1 - s - bN_1 - mN_1) + u(w(s,h)(1 - bN_2) - mN_2) + a(N_1 + N_2) \end{aligned}$$

Figure 24 shows a computed example with $a = 0.4, b = 0.175, \sigma = 0.7, m = 0.075$. Fertility first declines steeply with human capital, then rises. In addition, for parents with low AFLB ($N_1 > 0$), the fertility-human capital relationship is negative, while for parents with higher AFLB ($N_1 = 0$) it is positive.

### Concavity

#### Lemma 5

For $\sigma >0.5$, *U* in equation (2) is concave in $N_{1},N_{2}$ and *s*.

#### Proof

We examine the Hessian matrix of utility in each period. Note that period 1 utility is constant in $N_{2}$ and period 2 utility is constant in $N_{1}$. For period 1 the Hessian with respect to $N_{1}$ and *s* is:

$$\begin{aligned}&\left[ \begin{array}{cc} d^{2}u/dN_{1}^{2} &{} d^{2}u/dsdN_{1}\\ d^{2}u/dsdN_{1} &{} d^{2}u/ds^{2} \end{array}\right] \\&\quad =\left[ \begin{array}{cc} -\sigma b^{2} &{} -\sigma b\\ -\sigma b &{} -\sigma \end{array}\right] Y_{1}^{-\sigma -1} \end{aligned}$$

with determinant

$$\begin{aligned} (\sigma ^{2}b^{2}-\sigma ^{2}b^{2})Y_{1}^{-2\sigma -2}=0. \end{aligned}$$

Thus, first period utility is weakly concave. For period 2 with respect to $N_{2}$ and *s*, the Hessian is:

$$\begin{aligned}&\left[ \begin{array}{cc} d^{2}u/dN_{2}^{2} &{} d^{2}u/dsdN_{2}\\ d^{2}u/dsdN_{2} &{} d^{2}u/ds^{2}dN_{2} \end{array}\right] \\&\quad =\left[ \begin{array}{cc} -\sigma (bsh)^{2}Y_{2}^{-\sigma -1} &{} -(1-\sigma )bhY_{2}^{-\sigma }\\ -(1-\sigma )bhY_{2}^{-\sigma } &{} -\sigma [h(1-bN_{2}^{*})]^{2}Y_{2}^{-\sigma -1} \end{array}\right] \end{aligned}$$

with determinant

$$\begin{aligned}&(-\sigma (bsh)^{2}Y_{2}^{-\sigma -1})(-\sigma [h(1-bN_{2}^{*})]^{2}Y_{2}^{-\sigma -1})-(-(1-\sigma )bhY_{2}^{-\sigma })^{2}\\&\quad = \sigma ^{2}(bsh)^{2}[h(1-bN_{2}^{*})]^{2}Y_{2}^{-2\sigma -2}-(1-\sigma )^{2}(bh)^{2}Y_{2}^{-2\sigma }\\&\quad = \sigma ^{2}(bh)^{2}Y_{2}^{-2\sigma }-(1-\sigma )^{2}(bh)^{2}Y_{2}^{-2\sigma }\text {, using that }Y_{2}=(sh)(1-bN)\\&\quad = (bh)^{2}Y_{2}^{-2\sigma }(\sigma ^{2}-(1-\sigma )^{2}) \end{aligned}$$

which is positive if and only if $\sigma >0.5$. Thus, if $\sigma >0.5$ then the Hessian is negative definite and thus utility is concave; this combined with weak concavity of period 1, and linearity of $a(N_{1}+N_{2})$, shows that (2) is concave. $\square$

### Effect of *a*

#### Lemma 6

For $\sigma <1$, $d^2N^*/dadh > 0$, i.e. the effect of *a* increases at higher levels of human capital.

#### Proof

Differentiating (17) with respect to *a* gives

$$\begin{aligned} \frac{d^2N^*_t}{dadh} = -\frac{1}{b}\frac{1}{1-2\sigma }a^{-1}\left( \frac{a}{b}\right) ^{1/(1-2\sigma )}\frac{1-\sigma }{2\sigma -1}h^{(1-\sigma )/(2\sigma -1)-1} \end{aligned}$$

for $t=1,2$ when $N^*_1,N^*_2>0$. For $\sigma > 0.5$ this is positive.

When $N^*_1 = 0,N^*_2>0$, differentiating (21) with respect to *a* gives

$$\begin{aligned} \frac{d^2N_{2}^{*}}{dadh} = \frac{1}{b}\frac{1}{\sigma }a^{-1}\left( \frac{a}{b}\right) ^{-1/\sigma }\frac{1-\sigma }{\sigma }(s^{*}h)^{(1-2\sigma )/\sigma }(s^{*}+h\frac{ds^{*}}{dh}) \end{aligned}$$

which is $-a^{-1}/\sigma$ times (21) and hence is positive.

$$\square$$

## Footnotes

1 The selection effect $\beta$ equals *Cov*(*RLRS*, *PGS*)/*Var*(*PGS*). Since PGS are normalized to variance 1 and mean 0, this reduces to $Cov(RLRS, PGS) = E(RLRS\times PGS) - E(RLRS) E(PGS) = E(RLRS \times PGS)$. This is the polygenic score weighted by relative lifetime reproductive success, which is the average polygenic score in the next generation (Robertson 1966).

2 We also check for stabilizing and disruptive selection by estimating (1) with a quadratic term. Stabilizing selection selects for intermediate values, while disruptive selection selects for extreme values. In particular, we find disruptive selection for educational attainment polygenic scores: at higher values of these scores, the negative effect on fertility is smaller (Appendix Figure 10).

3 We use these weights throughout. All our qualitative results are robust if we run unweighted regressions. Appendix Table 2 shows results from alternative weighting schemes.

4 The same pattern holds if we analyse men and women separately (Appendix Fig.  11). We also directly compared selection effects between men and women (Appendix Fig. 9).

5 AFLB is unavailable for men.

6 Predictions 1-3 also hold in the one-period model with constant relative risk aversion. Our empirical results are actually stronger than prediction 5, in that correlations with fertility are *reversed* at higher AFLB. This prediction can be accommodated in the model if children have a money cost as well as a time cost (Appendix Figure 24).
