# Correction: Human Capital Mediates Natural Selection in Contemporary Humans

**DOI:** 10.1007/s10519-022-10110-1

**Published:** 2022-07-23

**Authors:** David Hugh-Jones, Abdel Abdellaoui

**Affiliations:** 1 School of Economics, University of East Anglia, Norwich, UK; 2 Department of Psychiatry, Amsterdam UMC, University of Amsterdam, Amsterdam, The Netherlands

## Correction to: Behavior Genetics 10.1007/s10519-022-10107-w

In the original version of this article, the statement of Human and Animal Rights was incorrectly given as

“All participants gave full informed written consent for participation in the UK Biobank. This study was performed in accordance with the criteria defined by the rules of the UK Biobank.”

but should be

“UK Biobank has received ethical approval from the National Health Service North West Centre for Research Ethics Committee (reference: 11/NW/0382).”

The original article has been corrected.

